# Lindsay & Blakiston’s Visiting List

**Published:** 1875-11

**Authors:** 


*Lindsay and Blakiston's Visiting List for* 1876.

We are again reminded of the approach of another year by the annual visit of this familiar pocket companion. Lindsay & Blakiston's visiting list is widely and favorably known in the profession as the best pocket record extant.

